# Facilitators and barriers of preconception care in women with inflammatory bowel disease and rheumatic diseases: an explorative survey study in a secondary and tertiary hospital

**DOI:** 10.1186/s12884-022-04560-y

**Published:** 2022-03-23

**Authors:** L. A. C. Admiraal, A. N. Rosman, R. J. E. M. Dolhain, R. L. West, A. G. M. G. J. Mulders

**Affiliations:** 1 Department of Obstetrics and Gynecology, Erasmus MC, University Medical Center Rotterdam, Room Sp-4469, PO Box 2040, 3000 CA Rotterdam, the Netherlands; 2 Department of Health Care Studies, Rotterdam University of Applied Sciences, Postbus, 25035 3001 HA Rotterdam, the Netherlands; 3 Department of Rheumatology, Erasmus MC, University Medical Center Rotterdam, PO Box 2040, 3000 CA Rotterdam, the Netherlands; 4 Department of Gastroenterology, Franciscus Gasthuis, PO Box 10900, BA 3004 Rotterdam, the Netherlands

**Keywords:** Preconception care, Chronic inflammatory disease, Inflammatory bowel disease, Rheumatic diseases, Obstetrics

## Abstract

**Background:**

Preconception care (PCC) is care prior to conception to optimize parental health, and health of the future child, through biomedical and behavioral changes. Providing PCC to all women with a wish to conceive will improve perinatal health. PCC is especially important for women with a chronic disease, such as inflammatory bowel disease (IBD) and rheumatic diseases (RD). At present PCC is not part of routine care for these women. The aim of this study is to identify facilitators and barriers on a patient and professional level regarding the provision of PCC in women with IBD and RD.

**Methods:**

An explorative survey study among women with IBD and RD, their treating physicians and obstetric professionals was performed. Patients with a wish to conceive, pregnant women or those with a recent pregnancy (< 1 year ago) visiting the outpatient clinic of a secondary and tertiary hospital and involved physicians and obstetric professionals were eligible.

**Results:**

A total of 71% of the IBD patients (*n* = 22/31) and 35% of the RD patients (*n* = 20/58) received a PCC consultation. PCC consultation was considered easy to enter, short in time and patients felt comfortable. Patients (71% IBD; 62% RD) preferred a personal PCC consultation with their disease specific specialist together with an obstetrician. Patients specifically wanted to receive information about the safety of medication use and disease activity following delivery. Of the included healthcare professionals 67% (*n* = 31) agreed PCC was applicable to their patients. Main barrier to providing PCC was lack of time and unavailability of professionals. In total 41% (*n* = 16) of obstetric professionals felt they had the knowledge and skills to provide PCC compared to 33% (*n* = 1) and 75% (*n* = 3) of gastroenterologists and rheumatologists, respectively.

**Conclusion:**

Lack of awareness and urgency for the effectuation can be seen as important barriers for implementation of PCC. Due to the explorative nature generalisation of the results is not allowed. In the future, adaptation of the curricula of healthcare professionals by implementing interventions for pregnancy planning and preparation will generate awareness. Modelling of the impact of PCC might prove useful in resolving the lack of urgency for PCC realization.

**Supplementary Information:**

The online version contains supplementary material available at 10.1186/s12884-022-04560-y.

## Background

Preconception care (PCC) is care prior to conception for all women or couples to optimize parental health and the health of the future child through biomedical and behavioral changes [1]. General PCC involves counselling, health promotion and risk reduction. General practitioners (GPs) and midwives are usually responsible for providing general PCC. Women receive advice about folic acid supplementation and optimal lifestyle [2–4]. For women suffering from chronic diseases specialized PCC should be offered by a gynecologist or disease specific specialist [5]. Patients should be counseled on how their disease can affect pregnancy and the risks of pregnancy associated with their disease [6].

Specialized PCC is especially important for women with chronic diseases, like inflammatory bowel disease (IBD) and rheumatic diseases (RD). IBD, Crohn’s disease (CD) and ulcerative colitis (UC), is often diagnosed at a reproductive age, 50% being diagnosed before the age of 35 [7]. The presence of active disease during pregnancy is one of the most important risk factors for adverse pregnancy outcomes like spontaneous miscarriage, preterm delivery and low birth weight [8, 9]. Ideally, the disease should be in remission at least 6 months before conception. Lack of knowledge results in incorrect beliefs regarding pregnancy. The study from Ellul et al. showed that > 60% of the patients believed IBD might lead to a complicated pregnancy and the disease itself or medication use could cause harm to the fetus [10]. Walldorf et al. showed that there is significantly more childlessness amongst women with IBD and stress the importance of qualified counselling as early as possible [11]. Providing PCC in women with IBD is associated with medication compliance, reduced disease relapse during pregnancy and a protective factor for having children with a low birth weight and therefore a better outcome of pregnancy [12].

PCC is also essential for women with rheumatoid arthritis (RA). Despite remission of this auto-immune disease during pregnancy, more than half of the RA patients experience active disease during the third trimester. Low disease activity before pregnancy is associated with low disease activity during pregnancy [13]. Whereas high disease activity in women with RA can be associated with infertility and low birthweight [14, 15]. Therefore, it is important that the disease is in remission before conception.

A study by Chakravaty et al. regarding family planning in women with systemic inflammatory diseases, like IBD and RD, reported that patients had a preference for receiving PCC from a gynecologist. A disease specific specialist should only be involved for the treatment of the underlying chronic condition. Only a minority of the disease specific specialists reported providing PCC to female patients of reproductive age [16]. Hence, women with systemic inflammatory diseases are in need of consistent information about disease specific pregnancy risks which their disease specific medical specialists do not routinely provide.

It is important to improve the quality of specialized PCC and to ensure that PCC is available for patients with a chronic inflammatory disease at risk for pregnancy complications due to their underlying disease. The aim of this explorative survey study is to identify facilitators and barriers on a patient and healthcare professional level regarding PCC in women with IBD and RD.

## Methods

### Study design

The PPCD (Pregnancy Preparation for women with Chronic Diseases) study is an explorative survey study (MEC-2016-368). Questionnaires on a patient and professional level (additional files 1, 2, 3, 4 and 5) were used to identify the facilitators and barriers of PCC. These questionnaires were based on earlier validated questions from the HP4All2 study (MEC–2015–182, Healthy pregnancy 4 All-2, subproject protocol C ‘interconception care’) which focused on the effectiveness of programmatic PCC and systematic antenatal risk assessment by introducing and offering PCC to women visiting family clinics in the Netherlands [17]. Women eligible were between 18 and 42 years of age and visiting the outpatient clinic for their specific disease (i.e. IBD and RD). Their actual wish to conceive was asked. They had an actual or nearby future (< 1 year) wish to conceive, were pregnant or recently gave birth (< 1 year ago). Patients with a poor understanding of the Dutch language were excluded.

Healthcare professionals (medical specialists, residents, house-officers, midwives and consultants) working in the collaborating departments (i.e. Gastroenterology, Rheumatology and Obstetrics & Gynecology) were also asked to participate.

### Study setting

Women were recruited from the departments of Gastroenterology and Obstetrics and Gynecology of a secondary hospital (Francisus Gasthuis & Vlietland, Rotterdam) and the departments of Gastroenterology, Rheumatology and Obstetrics and Gynecology of a tertiary hospital (Erasmus Medical Center, Rotterdam).

### Study procedure

Women who met the inclusion criteria were asked to participate in this study during an outpatient visit with their disease specific specialist. They received an information letter and an informed consent form. Only after informed consent did participating patients receive an e-mail with a link to the online questionnaire using Lime Survey, which is an online tool for sending out questionnaires to specific groups. The questionnaire was completely anonymous. Healthcare professionals also received an e-mail with a link to another anonymous, online questionnaire.

Both questionnaires included questions to identify baseline characteristics (client level: 27 questions; professional level: 5 questions); facilitators and barriers of PCC (client level: 25 questions; professional level: 9 questions); knowledge, attitude and actions towards PCC (client level: 13 questions; professional level: 9 questions) and the most ideal form of a preconception consultation (i.e. a personal or a skype consultation) (client level: 2 questions; professional level: 9 questions). In the analysis responses to questions were translated into facilitators or barriers. For example: ‘How easy/difficult is it for you to visit a PCC consultation?’. This question was answered with a four-point scale from ‘very easy’ to ‘very difficult’ whereby very difficult and difficult were seen as barriers and very easy and easy as facilitators. Facilitators and barriers were reported for the domains: personal, medical, organizational and financial. Knowledge was arbitrarily judged as adequate if a patient had a score of > 80% correct answers [18, 19].

### Statistical analysis

IBM SPSS Statistics version 24 was used to analyze data from both questionnaires. The baseline characteristics were determined for both patients and healthcare professionals. Frequency tables were used to analyze categorical and continuous data. Cross tables were used to report the results for knowledge, attitude and actions towards PCC. By analyzing the answers to both questionnaires, facilitators and barriers of PCC in women with IBD and RD were identified. All reported answers were considered as important and contributing to the questions and therefore included for analysis.

### Study sample

Every year approximately 90 patients meeting our inclusion criteria are referred to the outpatient clinic of the department of Rheumatology at the tertiary hospital. Further, approximately each year 40 eligible IBD patients are referred to the outpatient clinics of the departments of both (secondary and tertiary) participating hospitals. We planned for an inclusion period of 1 year.

## Results

### Results at a patient level

At the outpatient clinic 36 women with IBD and 63 women with RD were asked to participate. Eventually, the questionnaire was filled in by 31 women with IBD and 58 women with RD. Hence, the response rate was 92% for women with RD and 86% for women with IBD respectively. One response in the IBD group and six responses in the RD group were incomplete (missing 3.4%). The baseline characteristics of both groups are shown in Table 1. The majority of our patients were of Western origin. In total 55% (*n* = 17) of the women in the IBD group had CD and 42% (*n* = 13) had UC. The most common RD in our patients were RA (36%, *n* = 21) and ankylosing spondylitis (21%, *n* = 12). Regarding obstetric history, previous miscarriages were reported in 19% (*n* = 6) of women with IBD and in 32% (*n* = 18) of women with RD. At the time of enrollment 32% (*n* = 10) of the women in the IBD group and 45% (*n* = 26) of the women in the RD group were pregnant.

**Table 1:** Baseline characteristics women with IBD and RD

	Women IBD Frequency ***n*** = 31 (%)^a^	Women RD Frequency ***n*** = 58 (%)^a^
**Registration**		
Mean age, years	30 (25–38)	32 (27–41)
Country of origin		
Western	30 (97)	56 (97)
**Education and work**		
Understanding of the Dutch language	31 (100)	57 (98)
Education		
Low	12 (39)	21 (36)
Intermediate	12 (39)	24 (41)
High	7 (23)	12 (21)
Paid job	28 (90)	47 (81)
**Lifestyle and medical history**		
Smoking		
No	29 (94)	54 (93)
Smoking of partner		
No	24 (77)	51 (88)
Alcohol		
Yes	17 (55)	20 (35)
Drugs		
No	29 (94)	56 (97)
Folic-acid supplement		
Yes, daily	20 (65)	43 (74)
Inflammatory bowel disease		
Ulcerative colitis	13 (42)	0 (−)
Crohn’s disease	17 (55)	0 (−)
Other	1 (3)	0 (−)
Rheumatic disease		
Rheumatoid arthritis	0 (−)	21 (36)
Ankylosing spondylitis	0 (−)	12 (21)
Juvenile idiopathic arthritis	0 (−)	6 (10)
Psoriatic arthritis	0 (−)	10 (17)
Spondylarthropathy	0 (−)	6 (10)
Other	0 (−)	1 (2)
Time since diagnosis		
> 12 months	30 (97)	56 (97)
**Obstetric history**		
Mean number of pregnancies	2.0	2.0
Complicated pregnancy	1 (3.2)	11 (19)
Miscarriage		
Yes	6 (19)	18 (32)
No	25 (81)	37 (64)
One of the next problems during pregnancy		
Pre-term birth (< 37 weeks)	1 (3)	4 (7)
Birthweight < 2500 g	1 (3)	6 (10)
Birthweight > 4500 g	2 (7)	0 (−)
Congenital malformation	0 (−)	2 (3)
Perinatal asphyxia (Apgar 5 < 7)	4 (13)	4 (7)
Perinatal mortality		
No	31 (100)	58 (100)
Contraception		
Yes	14 (45)	14 (24)
**Actual wish to conceive**		
Pregnant at study entry	10 (32)	26 (45)
Within 3–12 months	16 (52)	16 (27)
Within 1–2 years	4 (13)	2 (3)
In 2 years or more	0 (−)	5 (9)
I don’t know	0 (−)	3 (5)

^a^due to missing answers (3.4%) the numbers do not always count up to 100%

Facilitators and barriers of PCC reported by patients with IBD and RD are shown in Table 2. On a personal level, multiple facilitators were found. Women in both groups said visiting a PCC consultation would be easy to enter ((90%, *n* = 28 IBD) and (86%, *n* = 50 RD)). The majority of women with IBD and RD felt comfortable visiting a PCC consultation and did not think it took too much time. An important reason to visit a PCC consultation was a good preparation for pregnancy which was reported by 74% (*n* = 23) of women with IBD and 74% (*n* = 43) of women with RD.

**Table 2:** Statements used to identify facilitators and barriers of PCC for women with IBD and RD

	Women IBD ***n*** = 31 (%)^a^ Facilitators Barriers		Women RD ***n*** = 58 (%)^a^ Facilitators Barriers	
**Personal**				
PCC before becoming pregnant is necessary.	28 (90)	2 (6)	48 (83)	4 (7)
Quit smoking would be easy.	22 (71)	8 (26)	42 (72)	10 (17)
Taking a folic-acid supplement every day would be easy.	27 (87)	3 (10)	50 (86)	2 (3)
Visit a PCC consult would be easy.	28 (90)	2 (7)	50 (86)	2 (3)
Having a conversation with a healthcare professional about your wish to conceive would be easy.	27 (87)	3 (10)	50 (86)	2 (3)
Visiting a PCC consult is allowed from my religion.	30 (97)	0 (−)	52 (90)	0 (−)
I do not fear negative responses from my partner or family when I visit a PCC consult.	29 (94)	1 (3)	49 (84)	0 (−)
My partner wants me to go to a PCC consult.	1 (3)	29 (93)	7 (12)	45 (78)
I would visit a PCC on advice of my family/friends.	0 (−)	30 (96)	3 (5)	49 (85)
I want information/a good preparation for a pregnancy.	23 (74)	7 (22)	43 (74)	9 (16)
A PCC consult gives me enough advantages.	28 (90)	2 (7)	49 (85)	3 (5)
I do not fear a PCC consult.	28 (90)	2 (7)	51 (88)	1 (2)
A PCC consult does not take me too much time and effort.	27 (87)	3 (10)	48 (83)	4 (7)
I can see reasons to visit a PCC consult.	30 (97)	0 (−)	49 (85)	3 (5)
**Medical**				
With PCC you know how to become healthy pregnant.	29 (93)	1 (3)	40 (69)	12 (21)
Having a child with a bad medical condition.	1 (3)	29 (93)	1 (2)	51 (88)
Having a pregnancy history with complications.	4 (13)	26 (83)	6 (10)	46 (80)
I would visit a PCC consult on advice of the disease specific specialist.	23 (74)	7 (22)	36 (62)	16 (28)
I would visit a PCC consult on advice of the midwife, gynecologist or general practitioner.	10 (32)	20 (64)	21 (36)	31 (54)
I would visit a PCC consult on advice of the youth healthcare physician.	1 (3)	29 (93)	4 (7)	48 (83)
**Organisation**				
PCC posters should be seen everywhere, like in the waiting room of the midwife/doctor.	20 (65)	10 (32)	35 (60)	17 (30)
**Financial**				
PCC consults should be available, for free, for everyone who wants to become pregnant.	28 (90)	2 (6)	49 (85)	3 (5)

^a^due to missing answers (3.4%) the numbers do not always count up to 100%

On a medical level, the course of a previous pregnancy and the healthcare professional referring for PCC can be seen as facilitators for PCC. In 4 out of 13 IBD and 6 out of 36 RD patients a previous pregnancy coincided with adverse events. The adverse pregnancy outcome corresponded with a positive attitude to PCC. Furthermore, the advice to visit a PCC consultation when given by a disease specific specialist was considered of higher value in both groups compared to receiving the advice for PCC from a gynecologist, midwife or GP*.*

On an organizational level the majority of the patients in both groups prefer to see PCC posters everywhere. On a financial level 90% (*n* = 28) of women with IBD and 85% (*n* = 49) of women with RD said PCC consultations should be available for free.

In both groups the preferred healthcare professional to provide PCC was the gynecologist and in second place from their disease specific specialist (gastroenterologist or rheumatologist). The most ideal form of a PCC consultation was a personal combined consultation from both their disease specific specialist and gynecologist (Table 3).

**Table 3:** Most ideal form of a preconception consultation regarding women with IBD or RD

Ideal form of a preconception consultation	Women IBD Frequency ***n*** = 31 (%)^a^	Women RD Frequency ***n*** = 58 (%)^a^
Personal consultation with the disease specific specialist	3 (10)	6 (10)
Personal consultation with the gynecologist	2 (7)	6 (10)
Personal consultation with the disease specific specialist and gynecologist	22 (71)	36 (62)
Skype consultation with the disease specific specialist	0 (−)	0 (−)
Skype consultation with the gynecologist	1 (3)	0 (−)
Skype consultation with the disease specific specialist and gynecologist	2 (7)	3 (5)

^a^due to missing answers (3.4%) the numbers do not always count up to 100%

Knowledge, attitude and actions towards general PCC of women with IBD and RD are described in an additional file (additional file 6). General knowledge on folic-acid supplementation was up to date in both groups as more than 80% understood the benefits of folic-acid supplementation. General knowledge on the effect of smoking on fertility was not up to date as less than 80% knew about the association with infertility.

The questionnaire also focused on the content of information patients would like to receive during a PCC consultation. Most of them wanted to receive information about medication use during pregnancy (97%, *n* = 30 IBD; 78%, *n* = 45 RD). Other important topics to be discussed were information about their disease after delivery and breastfeeding when using medication. In total 71% (*n* = 22) of the women with IBD and 35% (*n* = 20) of the women with RD visited a PCC consultation. They all felt that their questions were answered adequately and the majority found the consultation useful.

### Results at a professional level

The online questionnaire was sent to healthcare professionals from the involved departments. Responses were received from 39 professionals from the department of Obstetrics and Gynecology, three from the department of Gastroenterology and four from the department of Rheumatology. Not all questionnaires were filled in completely (missing 5.4%). The baseline characteristics of the respondents are shown in additional file 7.

Facilitators and barriers of PCC were identified on an organizational and personal level (Table 4). At an organizational level, shortage of healthcare professionals (62%, *n* = 24) and lack of time (54%, *n* = 21) to provide PCC were reported as barriers by respondents from the department of Obstetrics and Gynecology. Only one respondent from the department of Gastroenterology and none of the respondents from the department of Rheumatology reported lack of time. At a personal level 64% (*n* = 25) of the obstetric professionals agreed that from a healthcare professionals’ perspective, PCC was applicable to their patients which was in agreement with the respondents from the departments of Rheumatology and Gastroenterology.

**Table 4:** Statements used to identify facilitators and barriers of PCC for healthcare professionals

	Department Obstetrics / Gynecology ***n*** = 39 (85%)^a^		Department Gastroenterology ***n*** = 3 (6%)^a^		Department Rheumatology ***n*** = 4 (9%)^a^	
	Facilitators	Barriers	Facilitators	Barriers	Facilitators	Barriers
**Organisation**						
Having formal agreements about PCC in the department position paper.	9 (23)	24 (61)	3 (100)	0 (−)	3 (75)	1 (25)
Having an easy and fast access to information about providing PCC delivered by my department.	12 (31)	21 (54)	2 (67)	1 (33)	2 (50)	1 (25)
Getting enough time from my department to integrate PCC in my daily work.	12 (31)	21 (54)	2 (67)	1 (33)	3 (75)	0 (−)
Having enough healthcare professionals within my department to provide PCC.	9 (23)	24 (62)	2 (67)	1 (33)	2 (50)	1 (25)
Having access to ICT at my workplace (such as access to the internet or protocols) allows me to provide PCC.	20 (51)	13 (33)	2 (67)	1 (33)	3 (75)	0 (−)
**Personal**						
PCC fits in the way I work.	20 (51)	12 (31)	3 (100)	0 (−)	3 (75)	0 (−)
PCC is appropriate for my patients.	25 (64)	7 (18)	3 (100)	0 (−)	3 (75)	0 (−)
I expect that patients, in general, will be pleased when I give PCC to them.	25 (64)	7 (18)	3 (100)	0 (−)	3 (75)	0 (−)
I expect that patients, in general, cooperate when I give PCC to them.	23 (59)	9 (23)	3 (100)	0 (−)	3 (75)	0 (−)

^a^due to missing answers (5.4%) the numbers do not always count up to 100%

Knowledge, attitude and actions of healthcare professionals towards PCC are described in Table 5. From the Obstetrics and Gynecology department 41% of the respondents (*n* = 16) felt they had sufficient knowledge and skills to provide PCC. For the departments of Rheumatology and Gastroenterology this was the case in respectively 33% (*n* = 1) and 75% (*n* = 3).

**Table 5:** Statements used to identify knowledge, attitude and actions of healthcare professionals towards PCC

	Department of Obstetrics and Gynecology n = 39 (85%)		Department of Gastroenterology ***n*** = 3 (6%)^a^		Department of Rheumatology ***n*** = 4 (9%)^a^	
	Yes (%)	No (%)	Yes (%)	No (%)	Yes (%)	No (%)
**Knowledge up to date**						
Known with ZwangerWijzer.	26 (67)	8 (21)	1 (33)	2 (67)	2 (50)	2 (50)
Usage of ZwangerWijzer.	3 (8)	31 (80)	0 (−)	3 (100)	0 (−)	4 (100)
Known with R4U (Rotterdam Reproductive Risk Reduction).	25 (64)	9 (23)	0 (−)	3 (100)	0 (−)	4 (100)
Usage of R4U (Rotterdam Reproductive Risk Reduction).	11 (28)	23 (59)	0 (−)	3 (100)		
I have enough knowledge and skills to provide PCC.	16 (41)	16 (41)	1 (33)	2 (67)	3 (75)	0 (−)
PCC is too complicated for me to provide to patients.	0 (−)	32 (82)	1 (33)	2 (67)	1 (25)	2 (50)
**Attitude**	**Agree (%)**	**Disagree (%)**	**Agree (%)**	**Disagree (%)**	**Agree (%)**	**Disagree (%)**
I think it is a part of my job to provide PCC.	26 (67)	6 (15)	3 (100)	0 (−)	3 (75)	0 (−)
I think it is important to contribute to PCC.	27 (69)	5 (13)	3 (100)	0 (−)	3 (75)	0 (−)
**Actions (I would provide PCC to …)**	**Agree (%)**	**Disagree (%)**	**Agree (%)**	**Disagree (%)**	**Agree (%)**	**Disagree (%)**
All women (between 18 and 42 years of age) who visit to the clinic.	11 (28)	21 (54)	2 (67)	1 (33)	2 (50)	1 (25)
All women (between 18 and 42 years of age) who do not use contraception.	17 (44)	15 (39)	2 (67)	1 (33)	2 (50)	1 (25)
All women (between 18 and 42 years of age) with known risk factors for a next/future pregnancy.	30 (77)	2 (5)	2 (67)	1 (33)	2 (50)	1 (25)
All women (between 18 and 42 years of age) with questions about a next/future pregnancy.	31 (80)	1 (3)	3 (100)	0 (−)	3 (75)	0 (−)
All women (between 18 and 42 years of age) with a wish to conceive.	29 (74)	3 (8)	3 (100)	0 (−)	3 (75)	0 (−)
All men (between 18 and 42 years of age) who come to the clinic.	13 (33)	19 (49)	1 (33)	2 (67)	2 (50)	1 (25)

^a^due to missing answers (5.4%) the numbers do not always count up to 100%

**Fig. 1:**
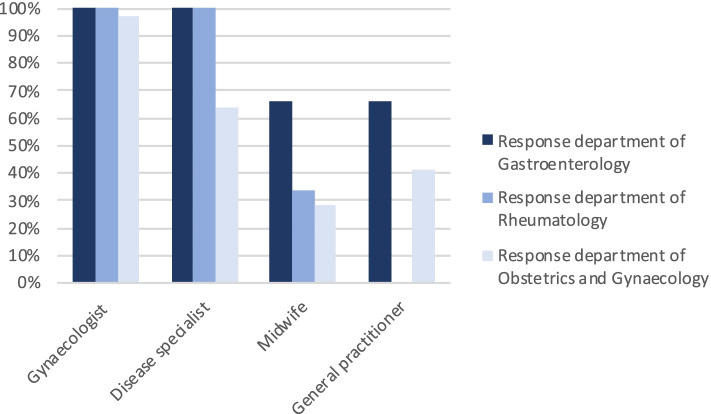
Healthcare professionals’ point of view: professionals suitable for PCC in women with IBD/RD. The profession of the healthcare professionals included in the answer to this question, depicted at the bottom of the figure, are respectively a gynecologist, a disease specialist, a midwife and a general practitioner. Ranking was performed according to a 5-point scale from ‘most certainly’ to ‘most certainly not’. Percentages were calculated based on number of respondents who filled in: ‘most certainly’ and ‘certainly’. The percentages reflect the suitability of the professional to provide PCC to women with IBD/RD according to the healthcare professionals, reflected as respondents, from respectively the department of Gastroenterology (dark blue), department of Rheumatology (blue) and department of Obstetrics and Gynecology (light blue)

Figure 1 (insert Fig. 1) shows which healthcare professionals were considered most suitable by healthcare professionals to provide PCC. In both women with IBD and RD the gynecologist and the disease specific specialist were mentioned as most suitable. Midwives and GPs were considered less suitable to provide PCC.

## Discussion

### Main findings

Facilitators and barriers have been reported on a patient and healthcare professional level. Patients seem to have a positive attitude towards PCC. They are interested in receiving information about the impact of their disease on pregnancy and vice versa. Furthermore, they prefer to receive information about their medication use during pregnancy and lactation. The professionals also have a positive attitude towards PCC as they think it is important to contribute to PCC and they see the need for PCC for their patients. Despite the positive attitude towards PCC on a client and professional level, realization of PCC remains difficult.

### Comparison to other studies

Studies have shown that women in general are interested in PCC and have a positive attitude towards PCC, however the uptake of PCC is low [3, 20]. The majority of women are hesitant about seeking PCC themselves, because they do not consider themselves part of the target group [3]. Our study found that 71% of the women in the IBD group experienced they had received a PCC consultation which is, crucial to mention, part of standard of care for women with IBD visiting the Department of Gastroenterology of our tertiary hospital. Only 35% of women with RD mentioned they received a PCC consultation. However, providing PCC, is standard of care in the outpatient department of RD of the tertiary hospital (additional file 6). These findings may be explained by variations in patient experience regarding discussion of the topic “pregnancy and pregnancy preparation” with their treating doctors.

Poels et al. reported several facilitators and barriers for general PCC. Some of the barriers identified were anxiety and fear of PCC, not being offered PCC and time and effort to visit a PCC consultation [20]. These were not identified as barriers in our study. The majority felt comfortable to visit a PCC consultation or thought such consultation did not take too much time. This could be due to the selected group of women who participated in our survey study.

The study from Chakravarty et al. showed that 32–56% of the gastroenterologists and rheumatologists routinely provided PCC to women of childbearing age [16]. Our study found that 67% of the gastroenterologists and 50% of the rheumatologists would provide PCC to all women who visit the clinic for routine care. However, the low number of included disease specific specialists of both departments in this study could affect these results.

Studies from Goossens et al. and M’Hamdi et al. about facilitators and barriers of PCC reported by healthcare providers mentioned more barriers than facilitators, including lack of knowledge and lack of time [21, 22]. This is partly in line with our findings. Some of the healthcare professionals reported to have lack of knowledge and time to provide PCC (Tables 4-5). Therefore, more information about PCC should be provided to healthcare professionals and more time should be created to implement PCC as part of standard-care.

A study regarding knowledge on folic acid supplementation showed that patients’ knowledge on this topic has increased over the years but remains limited. A study from Temel et al. showed an increase in knowledge from 30.7 to 36.8% in 2 years time [19]. In our study knowledge on folic acid supplementation was up to date in women with IBD and RD with more than 80% correct answers. On the contrary, knowledge of the effect of smoking on fertility was not up to date with less than 80% correct answers. These findings were supported by another study which showed that the majority of patients thought smoking did not affect fertility [23]. Several studies have shown that smoking by men and women is associated with delayed conception and adverse pregnancy outcomes [24–26]. The topics of folic acid supplementation and smoking should always be addressed during a PCC consultation.

In this study, we assessed patients’ knowledge using four questions from a validated Dutch questionnaire. However, it should be mentioned the CCPKnow (Crohn’s and Colitis Pregnancy Knowledge) score is available as a validated tool assessing patients’ knowledge [27]. Moreover, this educational tool has significantly improved IBD specific knowledge in patients.

### Relevance of the findings

Research has shown that women are hesitant about seeking PCC themselves and only a minority of primary caregivers in the Netherlands recommends PCC in the form of a dedicated consultation [3, 4]. This study shows that only 28% (*n* = 11) of the healthcare professionals from the department of Obstetrics and Gynecology would provide PCC to all women of childbearing age. The majority (74%, *n* = 34) would provide PCC to women with known risk factors or questions about a future pregnancy (Table 5). Hence, a considerable group of women with an increased risk of pregnancy complications who would potentially benefit from a PCC consultation, do not receive this form of care. Hence, following this survey study aiming to provide PCC to all women of childbearing age, we would like to suggest at least to disease specific specialists that mentioning PCC to their patients generating awareness of the existence and importance of this kind of care is an urgent matter. Further, GPs and midwives could play a key role during everyday practice stating the availability and importance of a PCC consultation during routine appointments. Lastly, considering the lack of knowledge mentioned by the professionals, professionals could receive training on the importance of PCC and the possibilities how to deliver PCC to all women of childbearing age.

PCC can be provided by various healthcare professionals. Our patients preferred to receive PCC from either a gynecologist or their disease specific specialist. A personal consultation with both specialists was reported as the ideal form of a PCC consultation (Table 3). We consider collaboration between the departments of Rheumatology and Obstetrics and Gynecology at the Erasmus MC as best practice, as healthcare professionals already participate in multidisciplinary consultations and preconceptional referral of patients with a wish to conceive. At the moment, the department of Obstetrics and Gynecology and the department of Gastroenterology have separate PCC consultations in the Erasmus MC. We would recommend closer collaboration between both departments to improve the quality of PCC. Sellinger et al. describe how to provide guidance for clinicians involved in the care of pregnant patients with IBD [5]. They mention that close collaboration and joint decision-making between IBD and obstetric teams is necessary for optimal care. These recommendations are supported by the findings of Kashkooli et al. who show that compared to gastroenterologists, gynecologists have inferior knowledge on IBD and reproduction [28]. Healthcare professionals from different hospitals in the South-West of The Netherlands have developed a IBD health care pathway using uniform working methods and protocols resulting in more optimal and efficient care for patients [29]. Such mutual collaborations will be helpful to overcome issues evolving due to the lack of knowledge between healthcare professionals from different backgrounds. In addition, recent studies from Atrash and Jack describe several evidence-based clinical interventions and guidelines for PCC implementation which can be useful to further optimize multidisciplinary PCC consultations [30–32].

Lack of awareness of PCC has been reported in different studies as a barrier [20–22]. One patient said she never knew about the existence of PCC and she would have liked to know about disease related complications before her first pregnancy. The majority of our women with IBD or RD reported that PCC posters should be used to alert women in, amongst others, the waiting room of the midwife/GP. Sijpkens et al. showed that visual information in waiting rooms could result in an increased number of women visiting a PCC consultation [33].

Research regarding the opportunities to improve preconception counseling for those not yet planning a pregnancy should be performed. Also we believe, to improve womens’ knowledge on pregnancy preparation all healthcare professionals should take responsibility and ask women of childbearing age for a future wish to conceive. In addition, Sijpkens et al. following an international expert meeting on interconception care, showed that as a start for the implementation of PCC one should start talking about a future wish to conceive with women of fertile age whenever you have the chance to do so [34].

It is important to state the financial organization of the healthcare system in the Netherlands differs when compared to other countries. To visit a medical specialist a financial fee is covered by the mandatory social health insurance plan, so PCC is covered for all women who are treated by a medical specialist. Due to the course of the medical treatment for IBD and RD a financial motive involved as an organizational barrier for our participants is absent. This is in contrast to the financial organization of the healthcare system in the United Kingdom (UK) where there is a dependence on government funding [35]. Further, according to the organization of community based specialty care systems such as in the US/Australia with insured medical specialist care at patients’ request implementation of PCC may also be hampered by a financial barrier.

Moreover, researchers from the UK proposed an annual report card using metrics from multiple routine data sources as to hold governments and other stakeholders to account for delivering interventions, focusing on a population level as well as on an individual level, to improve preconception health [35]. As part of planning and preparation for a pregnancy, at an individual level, interventions should focus on conversations about pregnancy intention for which the training curricula of health care professionals should be adapted [35]. Additionally, implementation of training programs for patients and for both health and non-health professionals is also suggested as a solution to facilitate effective PCC by others [36, 37].

### Strengths and limitations

One of the strengths of this study was the use of validated questions (based on the HP4All2 study) to identify facilitators and barriers of PCC [17]. Questionnaires were anonymous and easily accessible online. Questionnaires were filled in by patients and healthcare professionals, therefore facilitators and barriers could be identified on both levels. Furthermore, this study involved different areas of interest (department of Obstetrics and Gynecology, Gastroenterology and Rheumatology).

There were 46 healthcare professionals who filled in the questionnaire. Unfortunately, numbers of disease specialists included were low which can be seen as a limitation of the study. Due to the anonymity of the questionnaires, it was not possible to send personal reminders to fill in the questionnaires. However, in this study, we included a variety of healthcare professionals involved in PCC for women with IBD and RD, reflected by the qualification of the professionals as stated in additional file 7. The diversity of the included professionals adds to the strength of our study.

We were limited by the number of inclusions, as not all women visiting the outpatient clinics fulfilled our inclusion criteria. Also, we were dependent on the voluntary participation of women (with a chronic disease) further limiting our number and increasing our inclusion period beyond the calculated duration of the study.

Although the number of subjects did not meet our required sample size, the number of women included in our explorative study (58 women with RD and 31 women with IBD) is sufficient to identify facilitators and barriers of PCC for this specific group. The explorative nature provides a descriptive analysis and does not allow for generalisation of the results. The high response rate of the included participants (i.e. 92 and 86% for women with RD and IBD respectively) shows the included patients are highly motivated to share their opinion on facilitators and barriers regarding PCC which adds to the importance of this topic from their point of view. Yet, this study gives a good reflection of the way women with RD and IBD think about PCC and how they prepare themselves for a future pregnancy. Information on the exact status of the severity of the disease was not provided which can be seen as a limitation. Performing the study in a secondary and tertiary hospital the participants included reflect a representative population of women with IBD and RD.

We found that 71% of the patients with IBD visited a PCC consultation compared to 35% of the clients with RD. The study started in the RD group, later followed by the IBD group. In the first questionnaire we used a different word for a ‘preconception consultation’ which could have been misinterpreted by women. Moreover, this could be the reason for missing data on this question. During the study we substituted this word to a clearer word for ‘preconception consultation’ aiming for improved understanding of our survey question. In addition, despite the description of PCC in the questionnaire, it is possible women were not aware that PCC can be provided by various healthcare professionals such as for example their disease specific specialist. It is possible a larger number did receive PCC or were provided specific preconception information, which was not experienced as such, which can be seen as a limitation. Another explanation for the lower percentage of PCC in the RD group is the fact that many of the women visiting the outpatient clinic of Rheumatology were referred to the tertiary hospital after they had become pregnant subsequently lacking the opportunity to provide PCC in this center.

Finally, women reported they had to answer questions about a previous pregnancy, even though some of them had never been pregnant. It was not possible to skip the questions, so they answered that they did not have any problems during a previous pregnancy. This limitation for data interpretation was taken into account when constructing the results.

In this study, we included women with an actual or nearby future (< 1 year) wish to conceive, pregnant women or women who had recently given birth (< 1 year ago) which can be seen as a selection bias not including women without making plans for a pregnancy. However, answering the anonymous questionnaire some women reported a wish to conceive only after a period of 12 months or longer (Table 1). We consider these women not making actual plans also as important to answer the aim of our study due to their future child wish.

## Conclusion

Facilitators and barriers have been reported on a patient and healthcare professional level. Lack of awareness of the topic of PCC in patients and lack of urgency for the effectuation of PCC can be seen as important barriers for implementation of this form of fundamental care in routine clinical practice. Due to the explorative nature generalisation of the results is not allowed. In the future, it is important that both caregivers and patients come to see PCC as normal standard care before starting a pregnancy. This can be reached by informing women of childbearing age during each regular visit with a healthcare professional. Practically, conversations about pregnancy intention will result from adaptations of the training curricula of healthcare professionals implementing interventions for pregnancy planning and preparation. Economic modelling of the impact of PCC might prove useful in resolving lack of urgency by demonstrating the effectiveness and leveraging resources such as time required for PCC realization.

## Supplementary Information

**Additional file 1.** Questionnaire for women with IBD. Questions on a patient level (women with IBD) used to identify the facilitators and barriers of PCC.

**Additional file 2.** Questionnaire for women with RD. Questions on a patient level (women with RD) used to identify the facilitators and barriers of PCC.

**Additional file 3.** Questionnaire for healthcare professionals involved in the treatment of women with IBD. Questions on a healthcare professional level (treating women with IBD) used to identify the facilitators and barriers of PCC.

**Additional file 4.** Questionnaire for healthcare professionals involved in the treatment of women with RD. Questions on a healthcare professional level (treating women with RD) used to identify the facilitators and barriers of PCC.

**Additional file 5.** Questionnaire for obstetric healthcare professionals. Questions on a healthcare professional level (obstetric professionals) used to identify the facilitators and barriers of PCC.

**Additional file 6.** Questions used to identify knowledge, attitude and actions of women with IBD and RD towards PCC.

**Additional file 7.** Baseline characteristics healthcare professionals.

## Data Availability

The datasets used and/or analyzed during the current study are available from the corresponding author on reasonable request.

## Abbreviations

CD: Crohn’s disease
UC: Ulcerative colitis
Erasmus MC: Erasmus Medical Center
GP: General practitioner
IBD: Inflammatory bowel disease
PCC: Preconception care
PPCD: Pregnancy Preparation of women with Chronic Diseases
RA: Rheumatic arthritis
RD: Rheumatic diseases
