# Supplementary material for: Facilitators and barriers of preconception care in women with inflammatory bowel disease and rheumatic diseases: an explorative survey study in a secondary and tertiary hospital
Source: BMC Pregnancy Childbirth. 2022 Mar 23;22:238. doi: 10.1186/s12884-022-04560-y (PMC8944158; doi:10.1186/s12884-022-04560-y)
Supplement: Supplementary file 2 — Additional file 2. Questionnaire for women with RD. Questions on a patient level (women with RD) used to identify the facilitators and barriers of PCC. [file 12884_2022_4560_MOESM2_ESM.docx]

**Additional file 2.** Questionnaire for women with RD
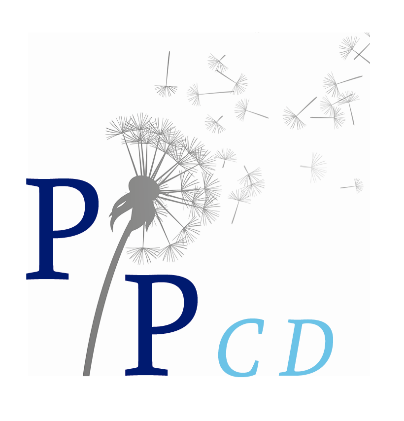

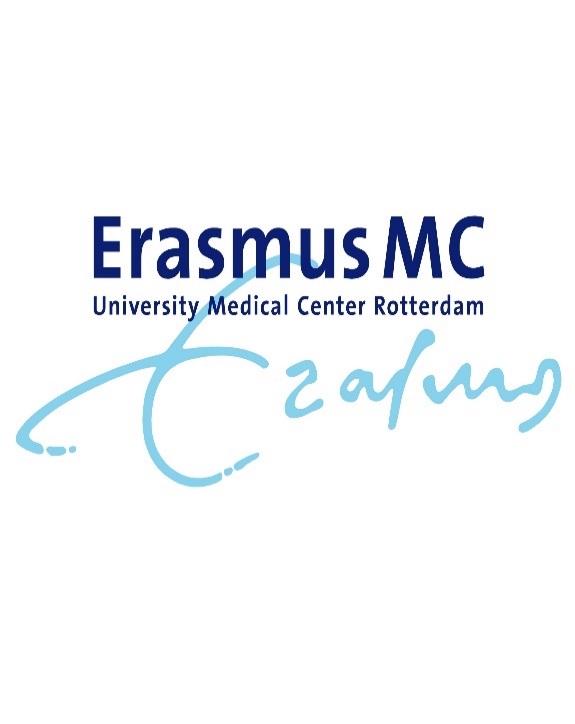
.

***P****regnancy* ***P****reparation for women with* ***C****hronic* ***D****iseases*

Women with chronic auto-immune diseases, like rheumatoid arthritis, have a higher risk on adverse pregnancy outcomes than healthy women. Furthermore, there are several medicines which are harmful during pregnancy and should be changed preconceptionally. It is important to receive good advices before, during and after pregnancy. Therefore, we will explore your experiences with these advices through this questionnaire.

It will take about 15 minutes to complete the questionnaire.

There is space for your comments at the end of the questionnaire.

Your answers will be processed confidential and anonymously.

We will not ask your name.

## General information – about you

1. What is your age? ­­­­­­­­­­______________________

2. Can you indicatie in which country you, your mother and your father were born?

|  | You | Your mother | Your father |
| --- | --- | --- | --- |
| The Netherlands | □ | □ | □ |
| Suriname | □ | □ | □ |
| Antilles/ Aruba | □ | □ | □ |
| Morocco | □ | □ | □ |
| Turkey | □ | □ | □ |
| Oost-Europa | □ | □ | □ |
| Cape Verde | □ | □ | □ |
| Indonesia / the Moluccas | □ | □ | □ |
| Other |  |  |  |

3. Which population group do you consider yourself part of?

□ Dutch □ Turkish

□ Suriname-Creole □ Kurdish

□ Suriname-Hindustani □ Moroccan: Berbers

□ Suriname-Javan □ Moroccan: Arabic

□ Suriname - other: □ Indonesian/Moluccan

□ Antillean/Aruban □ Polish

□ Other:

## General information – education and work

4. Are you bale to read Dutch?

□ no □ yes, without problems

□ yes, a little bit

5. If you visit a healthcare provider, are you able to understand what he/she tells you?

□ no □ often

□ rarely □ always

□ sometimes

6. What is the highest level of education that you have completed?

□ Primary school

□ Special education, learning difficulties (children with learning difficulties [MLK], children with severe learning difficulties [ZMLK], severely maladjusted children [ZMOK])

□ Preparatory vocational education (VBO)/preparatory vocational secondary education (VMBO) basic vocational or advanced vocational track

□ Junior general secondary education (MAVO)/preparatory vocational secondary education (VMBO) combined or theoretical track

□ Senior general secondary education (HAVO)

□ Pre-university education (VWO)

□ Senior secondary vocational education (MBO)

□ Higher professional education (HBO)

□ University education (university/post HBO)

□ Other __________________________

7. Do you have a paid job at the moment?

□ no □ yes

8. Do you have financial problems or debts which are difficult to pay off at the moment?

□ no □ yes

## General information – lifestyle and medical

9. Do you smoke?

□ yes, every day

□ yes, but only now and again

□ no, I have never smoked: go to question 11

□ no, but I did in the past: go to question 11

10. How much do you smoke on a normal day?

□ ≤ 5 cigarettes per day

□ 6-10 cigarettes per day

□ 11-20 cigarettes per day

□ ≥ 20 cigarettes per day

□ I smoke cigars

11. Does your partner smoke?

□ yes

□ no

□ I don’t have a partner

12. Do you drink alcohol?

□ yes, every day

□ yes, but only now and again

□ no, I have never drunk alcohol

□ no, but I did in the past

13. Do you use drugs?

□ yes, every day

□ yes, but only now and again

□ no, I have never used drugs

□ no, but I did in the past

14. Are you taking folic acid tablets at the moment?

□ yes, every day

□ yes, but only now and again

□ no

15. Which rheumatic disease do you have?

□ Rheumatoid arthritis

□ Ankylosing spondylitis

□ Systemic lupus erythematosus

□ Juvenile idiopathic arthritis

□ Psoriatic arthritis

□ Spondylarthropathy

□ I don’t know

□ Other_______________________

16. How long have you been diagnosed with this rheumatic disease?

□ ≤ 12 months □ > 12 months

17. Are you using any prescription medicines (prescribed by a doctor) at the moment?

□ yes, *(you can choose multiple answers)*

□ Methotrexate (Ledertrexate)

□ Plaquenil (Hydrochloroquine)

□ Salazopyrine (Sulfasalazine)

□ Prednisone/prednisolone

□ Enbrel (Etanercept)

□ Humira (Adalumimab)

□ Remicade (Infliximab) □ Cimzia (Certrolizumab)

□ Simponi (Golimumab)

□ Orencia (Abatacept)

□ Mabthera (Rituximab)

□ Kineret (Anakinra)

□ RoActemra (Tocilizumab)

□ Stelara (Ustekinumab)

□ Cuprimine/Depan (D-penicillamine)

□ Imuran (Azathioprine)

□ Endoxan (Cyclofosfamide)

□ Arava (Leflunomide)

□ Neoral/Sandimmune (Ciclosporine)

□ Gold Therapy (Tauredon)

□ I do not know whether I am taking any medicines

□ no

□ other, ___________________

18. Do you take any medicines that you can buy without a prescription (for example at a chemist) at the moment?

□ yes, (you can choose multiple answers)

□ vitamin pills (suitable for pregnancy and if you want to become pregnant)

□ painkillers

□ medicines for colds

□ Tranquillisers or sleeping pills

□ homeopathic medicines

□ I do not know whether I am taking any medicines□ no

□ other, ___________________

## Pregnancy and wish to conceive

19. How many times have you been pregnant?

_____ times

20. Did you have any problems in your previous pregnancy/pregnancies? Such as diabetes, high blood pressure or pre-eclampsia?

□ yes

□ no

□ other, _____

21. Have you ever had a miscarriage?

□ yes, once

□ yes, twice

□ yes, more than 2

□ no

22. Have you ever had an abortion?

□ yes

□ no

23. Did you experience one of the following problems in your previous pregnancy/pregnancies?

23.1 Was (one of) your child(ren) born too early? Before about 8.5 months (37 weeks) of pregnancy? □ yes

□ no

23.2 Was (one of) your child(ren) born weighing less than 2,500 grams? □ yes

□ no

23.3 Was (one of) your child(ren) born weighing more than 4,500 grams?□ yes

□ no

23.4 Does (one of) your child(ren) have a birth defect? □ yes

□ no

23.5 Did (one of) your child(ren) have a poor start following the birth (for example, blue or limp) for which the paediatrician had to assist or the baby needed help with breathing?

□ yes

□ no

23.6 Have you ever had a child that died in the period surrounding the birth?

□ no

□ yes, before birth

□ yes, during birth

□ yes, in the first week after birth

□ yes, later than the first week after birth

24. Do you or your partner use contraceptives? (like contraceptive pills or condoms)

□ no

□ yes

25. When do you hope or expect to become pregnant again?

□ I am pregnant

□ Between now and 3 months o Within 3 to 6 months

□ Within 6 months to a year

□ Within one to 2 years

□ In 2 years or more

□ I do not know yet when I want to become pregnant again

□ I have been advised not to become pregnant again (for the time being)

□ I do not want to become pregnant again

## Pregnancy and wish to conceive

**Knowledge**

There are issues that can increase the chances of a healthy start to the pregnancy and the chances of a healthy baby. There are also issues that can form a risk to the pregnancy and the baby.

26. What do you think is true and false?

26.1 If you smoke, it will take longer to become pregnant.

□ True

□ False

□ I do not know

26.2 If you smoke during the pregnancy, the risk of premature birth is higher

□ True

□ False

□ I do not know

26.3 Folic acid is good for the baby’s growth and development

□ True

□ False

□ I do not know

26.4 The best time to start taking folic acid tablets is as soon as you have become pregnant

□ True

□ False

□ I do not know

**Your ideas about a PCC consultation**

The purpose of a PCC consultation is to inform you about the best ways to prepare yourself for a pregnancy and how you can start healthy with a pregnancy. A PCC consultation can be given by a midwife, general practitioner or gynecologist. Disease specific specialists also provide information about a good pregnancy preparation.

We would like to know your opinion on a PCC consultation.

27. What kind of information do you want to receive during a PCC consultation? (you can choose multiple answers)

□ information about becoming pregnant

□ information about pregnancy and my medicines

□ information about lactation and my medicines

□ information about my disease postpartum

□ information about my disease and contraception

28. The aim of a PCC consultation is to have the healthiest possible start to the pregnancy. We would like to know what you think about it.

28.1 A PCC consultation before the start of a pregnancy is not necessary.

□ I agree entirely

□ I agree

□ Neutral

□ I disagree

□ I disagree entirely

28.2 A PCC consultation must be advertised everywhere on posters. For example, at the GP, the midwife, the Child Health Clinic, at tram/bus stops, in shops and in public buildings.

□ I agree entirely

□ I agree

□ Neutral

□ I disagree

□ I disagree entirely

28.3 A PCC consultation should be accessible free of charge for everyone who wants to become pregnant.

□ I agree entirely

□ I agree

□ Neutral

□ I disagree

□ I disagree entirely

28.4 If you visit a PCC consultation, then you will know how to achieve a healthy pregnancy

□ I agree entirely

□ I agree

□ Neutral

□ I disagree

□ I disagree entirely

**Own effort**

29. You may already receive advice from a midwife, doctor or nurse before the pregnancy. We would like to know how difficult you find it to follow this advice.

29.1If you (were to) smoke: what would it be like for you to stop smoking?

□ Very difficult

□ Fairly difficult

□ Fairly easy

□ Very easy

29.2 How easy/difficult is it for you to take a pill (folic acid) every day?

□ Very difficult

□ Fairly difficult

□ Fairly easy

□ Very easy

29.3 How easy/difficult is it for you to visit a PCC consultation?

□ Very difficult

□ Fairly difficult

□ Fairly easy

□ Very easy

29.4 How easy/difficult is it for you to discuss your desire for a child with a healthcare provider?

□ Very difficult

□ Fairly difficult

□ Fairly easy

□ Very easy

**Barriers to attending a PCC consultation**

30. We would like to know why you might prefer not to go to a PCC consultation.

30.1 It takes too much time and effort to visit a PCC consultation.

□ I agree entirely

□ I agree

□ Neutral

□ I disagree

□ I disagree entirely

30.2 I fear going to a PCC consultation

□ I agree entirely

□ I agree

□ Neutral

□ I disagree

□ I disagree entirely

30.3 I will not advantage enough from going to a PCC consultation.

□ I agree entirely

□ I agree

□ Neutral

□ I disagree

□ I disagree entirely

30.4 I fear for negative reactions from my husband or family if I go to a PCC consultation.

□ I agree entirely

□ I agree

□ Neutral

□ I disagree

□ I disagree entirely

30.5 My religion or personal beliefs say that I should not go to a PCC consultation.

□ I agree entirely

□ I agree

□ Neutral

□ I disagree

□ I disagree entirely

**Reasons to visit a PCC consultation**

31. What is the most important reason for deciding that you would visit a PCC consultation? You can choose multiple answers.

□ I would like to receive information/I want to prepare for a subsequent pregnancy

□ Following advice from the youth healthcare physician

□ Following advice from the midwife, gynecologist or GP

□ Following advice from the disease specific specialist (rheumatologist)

□ My partner wants me to go

□ Following advice from my family/friends

□ The outcome of a previous pregnancy was not what I wanted

□ I have a child with a condition

□ I see no reason to go

□ Other: ­­________________________

**Statements about the baby’s health and illness**

32. Below we want to find out what you think you can do yourself to have a healthy baby

32.1 There are things that I can do before I become pregnant to ensure that my baby is born healthy

□ I agree entirely

□ I agree

□ Neutral

□ I disagree

□ I disagree entirely

32.2 There is a lot that I can do to ensure that my baby is born healthy

□ I agree entirely

□ I agree

□ Neutral

□ I disagree

□ I disagree entirely

32.3 There is nothing that I can do to ensure that my baby is born healthy

□ I agree entirely

□ I agree

□ Neutral

□ I disagree

□ I disagree entirely

**Your experience with PCC consultation**

33. Have you ever visited a PCC consultation

□ yes, go to question 33.1

□ no, go to question 34

33.1 I was given the feeling I could discuss anything.

□ I agree entirely

□ I agree

□ Neutral

□ I disagree

□ I disagree entirely

33.2 I was given the option to decide for myself whether or not to go to a PCC consultation.

□ I agree entirely

□ I agree

□ Neutral

□ I disagree

□ I disagree entirely

33.3 My privacy was respected.

□ I agree entirely

□ I agree

□ Neutral

□ I disagree

□ I disagree entirely

33.4 My questions were begin answered.

□ I agree entirely

□ I agree

□ Neutral

□ I disagree

□ I disagree entirely

33.5 I could visit the consultation at the moment that I wanted.

□ I agree entirely

□ I agree

□ Neutral

□ I disagree

□ I disagree entirely

33.6 I could visit the PCC consultation with the healthcare professional I wanted.

□ I agree entirely

□ I agree

□ Neutral

□ I disagree

□ I disagree entirely

33.7 The healthcare professional was well informed.

□ I agree entirely

□ I agree

□ Neutral

□ I disagree

□ I disagree entirely

33.8 The PCC consultation was of value to me.

□ I agree entirely

□ I agree

□ Neutral

□ I disagree

□ I disagree entirely

34. What is your ideal form of a preconception consultation?

□ Personal consultation with the disease specific specialist

□ Personal consultation with the gynecologist

□ Personal consultation with the disease specific specialist and gynecologist

□ Skype consultation with the disease specific specialist

□ Skype consultation with the gynecologist

□ Skype consultation with the disease specific specialist and gynecologist

**Your experience with the healthcare professionals**

35. Will you choose for a gynecologist at the Erasmus Medical Center when you become pregnant?

□ yes, go to question 36

□ no, go to question 37

36. Yes (you can choose multiple answers):

□ this is close to my home

□ on advice of disease specific specialist

□ on advice of the midwife, gynecologist of general practitioner

□ on advice of the youth healthcare physician

□ on advice of my family/friends

□ other _________________

37. No (you can choose multiple answers):

□ this is far away from my home

□ on advice of disease specific specialist

□ on advice of the midwife, gynecologist of general practitioner

□ on advice of the youth healthcare physician

□ on advice of my family/friends

□ other _________________

38. How often do you receive the same advice from different healthcare professionals?

□ Never

□ Sometimes

□ Regularly

□ Most of the time

□ Always

39. How often do you receive different advices from different healthcare professionals?

□ Never

□ Sometimes

□ Regularly

□ Most of the time

□ Always

40. How often do you have to retell your story to different healthcare professionals?

□ only to the rheumatologist

□ only to the rheumatology consultant

□ to the rheumatologist and the rheumatology consultant

□ to every healthcare professional

41. Do you find it annoying to have to retell your story?

□ yes

□ a little

□ no

42. Which healthcare professional would you prefer to go to for advice on a (next) pregnancy? *(make a list in the order of your preference with your first preference at the top)*

□ gynecologist

□ rheumatologist

□ rheumatology consultant

□ general practitioner

□ midwife

□ pediatrician

□ youth healthcare physician

□ youth healthcare nurse

43. How would you prefer to receive advice about your wish to conceive?

*(make a list in the order of your preference with your first preference at the top)*

□ skype

□ telephone

□ personal consultation

□ public website

□ e-mail

□ other _______________

| **Space for comments about the questionnaire:**  This is not meant for medical questions.  If you like, you can write down your contact here. |
| --- |
|  |

**This is the end of the questionnaire.**

**Thank you for completing the questionnaire.**
