# Supplementary material for: Facilitators and barriers of preconception care in women with inflammatory bowel disease and rheumatic diseases: an explorative survey study in a secondary and tertiary hospital
Source: BMC Pregnancy Childbirth. 2022 Mar 23;22:238. doi: 10.1186/s12884-022-04560-y (PMC8944158; doi:10.1186/s12884-022-04560-y)
Supplement: Supplementary file 5 — Additional file 5. Questionnaire for obstetric healthcare professionals. Questions on a healthcare professional level (obstetric professionals) used to identify the facilitators and barriers of PCC. [file 12884_2022_4560_MOESM5_ESM.docx]

**Additional file 5.** Questionnaire for obstetric healthcare professionals.


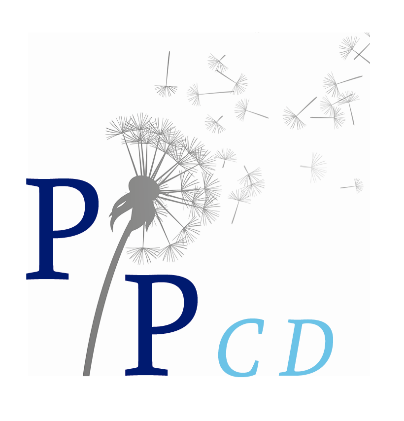

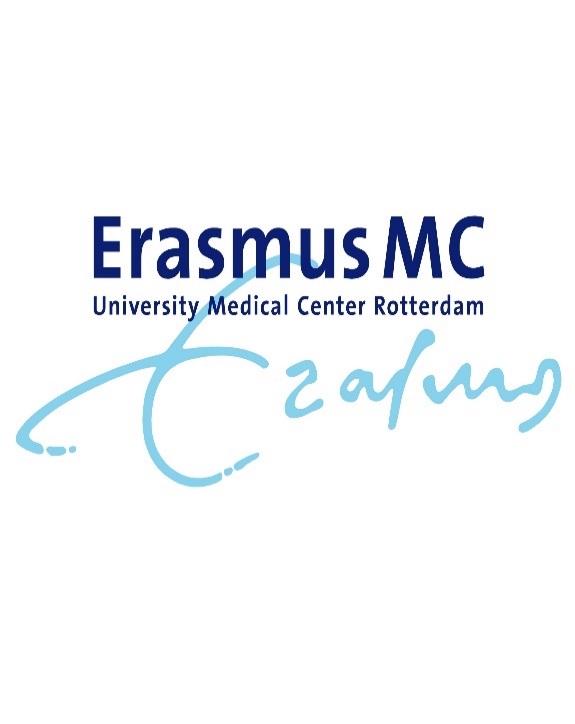


***P****regnancy* ***P****reparation for women with* ***C****hronic* ***D****iseases*

Women with chronic auto-immune diseases, like rheumatoid arthritis or inflammatory bowel disease (IBD), have a higher risk on adverse pregnancy outcomes than healthy women. Furthermore, there are several medicines which are harmful during pregnancy and should be changed preconceptionally. It is important that these women receive proper care preconceptionally up to the postpartum period. With this questionnaire want to explore how preconception care (PCC) is arranged and how you think about providing PCC to these women.

It will take about 10 minutes to complete the questionnaire.

There is space for your comments at the end of the questionnaire.

Your answers will be processed confidential and anonymously.

We will not ask your name.

## General information

1. What is your age? ___________________________
2. What is your gender? □ male

□ female

1. What is your specialism? □ Gynecologist-

perinatologist

□ Gynecologist-reproductive medicine

□ Resident gynecology

□ Midwife

□ Fertility doctor

1. How many years are you BIG registered? □ < 1 year

□ 1 - 5 years

□ 5 - 10 years

□ 10 - 15 years

□ 15 - 20 years

□ > 20 years

1. How many years are you registered as specialist? □ < 1 year

□ 1 - 5 years

□ 5 - 10 years

□ 10 - 15 years

□ 15 - 20 years

□ > 20 years □ inapplicable

## Minimal necessary care

PCC is all information, giving to your patients ant their partners, focused on getting pregnant healthy. This includes flyers about healthy pregnancy, referral to a website, referral to a specialized PCC consult, and so on.

1. Which form of PCC you think is minimal necessary to provide to your patients?

□ general information

□ specific information (i.e. disease and medication)

□ general and specific information (i.e. disease and medication)

□ general and specific information + referral to gynecologist for PCC consultation

□ in case of a wish to conceive direct referral to gynecologist

□ other: _______________________

## Instruments

ZwangerWijzer is an online screening tool which explores the risks focused on a healthy pregnancy. With the results, filled in by your patients, you can give specific information as a PCC consultation. The questionnaire can be filled in by patients as well as their partners.

The R4U (Rotterdam Reproductive Risks Reduction scorecard) is a screening tool for screening on six domains: psycho-social, care- and lifestyle related, medic and obstetric risk factors amongst pregnant women. It can be filled in during a first pregnancy consult at the midwife or gynecologist (before 12 weeks of gestation). R4U is developed for early detection and prevention of medical and non-medical risk factors.

1. Are you known with ZwangerWijzer?

□ yes, go to question 8

□ no, go to question 9

1. With how many patients you use ZwangerWijzer?

□ none

□ a minority

□ half

□ a majorty

□ everyone

1. Are you known with R4U?

□ yes, go to question 10

□ no, go to question 11

1. With how many patients you use R4U?

□ none

□ a minority

□ half

□ a majorty

□ everyone

## Access

1. My department has formal agreements about PCC in the department position paper.

□ yes

□ no

□ I don’t know

1. My department has easy and fast access to information about providing PCC.

□ Strongly agree

□ Agree

□ No opinion

□ Disagree

□ Strongly disagree

1. My department gives me enough time to integrate PCC in my daily work.

□ Strongly agree

□ Agree

□ No opinion

□ Disagree

□ Strongly disagree

1. My department has enough healthcare professionals to provide PCC.

□ Strongly agree

□ Agree

□ No opinion

□ Disagree

□ Strongly disagree

1. My department has access to ICT at my workplace (such as access to internet or protocols) which allows me to provide PCC.

□ Strongly agree

□ Agree

□ No opinion

□ Disagree

□ Strongly disagree

## Knowledge and job’s perspective

1. PCC is too complicated for me to provide.

□ Strongly agree

□ Agree

□ No opinion

□ Disagree

□ Strongly disagree

1. PCC fits in the way I work.

□ Strongly agree

□ Agree

□ No opinion

□ Disagree

□ Strongly disagree

1. I think it is important to contribute to PCC.

□ Strongly agree

□ Agree

□ No opinion

□ Disagree

□ Strongly disagree

1. I think it is part of my job to provide PCC.

□ Strongly agree

□ Agree

□ No opinion

□ Disagree

□ Strongly disagree

1. I expect that patients will generally be satisfied if I provide PCC

□ Strongly agree

□ Agree

□ No opinion

□ Disagree

□ Strongly disagree

21. I expect that patients will generally cooperate if I provide PCC.

□ Strongly agree

□ Agree

□ No opinion

□ Disagree

□ Strongly disagree

## Provide PCC

24. I would provide PCC to …

24.1 All women (between 18-42 years of age) who visit to the clinic.

□ Very certainly not

□ Certainly not

□ Maybe

□ Certainly yes

□ Very certainly yes

24.2 All women (between 18-42 years of age) who do not use contraception.

□ Very certainly not

□ Certainly not

□ Maybe

□ Certainly yes

□ Very certainly yes

24.3 All women (between 18-42 years of age) with known risk factors for a next/future pregnancy.

□ Very certainly not

□ Certainly not

□ Maybe

□ Certainly yes

□ Very certainly yes

24.4 All women (between 18-42 years of age) with questions about a next/future pregnancy.

□ Very certainly not

□ Certainly not

□ Maybe

□ Certainly yes

□ Very certainly yes

24.5 All women (between 18-42 years of age) with a wish to conceive.

□ Very certainly not

□ Certainly not

□ Maybe

□ Certainly yes

□ Very certainly yes

24.6 All men (between 18-42 years of age) who come to the clinic.

□ Very certainly not

□ Certainly not

□ Maybe

□ Certainly yes

□ Very certainly yes

25. Which of the following healthcare professionals do you think are suitable to deliver PCC to women with rheumatic diseases or IBD?

25.1 General practitioner.

□ Very certainly not

□ Certainly not

□ Maybe

□ Certainly yes

□ Very certainly yes

25.2 Midwife

□ Very certainly not

□ Certainly not

□ Maybe

□ Certainly yes

□ Very certainly yes

25.3 Gynecologist.

□ Very certainly not

□ Certainly not

□ Maybe

□ Certainly yes

□ Very certainly yes

25.4 Rheumatologist. 25.4 Gastroenterologist

□ Very certainly not □ Very certainly not

□ Certainly not □ Certainly not

□ Maybe □ Maybe

□ Certainly yes □ Certainly yes

□ Very certainly yes □ Very certainly yes

25.5 Other ________________

| **Space for comments about the questionnaire:** |
| --- |
|  |

**This is the end of the questionnaire.**

**Thank you for completing the questionnaire.**
