# Supplementary material for: Facilitators and barriers of preconception care in women with inflammatory bowel disease and rheumatic diseases: an explorative survey study in a secondary and tertiary hospital
Source: BMC Pregnancy Childbirth. 2022 Mar 23;22:238. doi: 10.1186/s12884-022-04560-y (PMC8944158; doi:10.1186/s12884-022-04560-y)
Supplement: Supplementary file 6 — Additional file 6. Questions used to identify knowledge, attitude and actions of women with IBD and RD towards PCC. [file 12884_2022_4560_MOESM6_ESM.docx]

**Additional file 6.** Questions used to identify knowledge, attitude and actions of women with IBD and RD towards PCC.

| **Knowledge up to date** | **Women IBD**  **Yes**  **n = 31 (%)*** | **No**  **n = 31 (%)*** | **Women RD**  **Yes**  **n = 58 (%)*** | **No**  **n = 58 (%)*** |
| --- | --- | --- | --- | --- |
| Folic-acid supplementation | >80% | - | > 80% | - |
| Smoking | - | <80% | - | < 80% |
| **Attitude** | **Agree**  **n = 31 (%)*** | **Disagree**  **n = 31 (%)*** | **Agree**  **n =58 (%)*** | **Disagree**  **n =58 (%)*** |
| There are things I can do before I become pregnant to deliver my baby healthy | 27 (87) | 3 (10) | 47 (81) | 5 (9) |
| There are lots of things I can do to deliver my baby healthy | 26 (84) | 4 (13) | 47 (81) | 5 (9) |
| There is nothing that I can do to deliver my baby healthy | 5 (16) | 25 (81) | 2 (3) | 50 (86) |
| **Actions** | **Yes**  **n = 31 (%)*** | **No**  **n = 31 (%)*** | **Yes**  **n = 58 (%)*** | **No**  **n = 58 (%)*** |
| Have you ever visited a preconception consult? | 22 (71) | 8 (26) | 20 (35) | 32 (55) |

* due to missing answers (3.4%) the numbers do not always count up to 100%
