# Supplementary material for: Facilitators and barriers of preconception care in women with inflammatory bowel disease and rheumatic diseases: an explorative survey study in a secondary and tertiary hospital
Source: BMC Pregnancy Childbirth. 2022 Mar 23;22:238. doi: 10.1186/s12884-022-04560-y (PMC8944158; doi:10.1186/s12884-022-04560-y)
Supplement: Supplementary file 7 — Additional file 7. Baseline characteristics healthcare professionals. [file 12884_2022_4560_MOESM7_ESM.docx]

|  | **Department Obstetrics /Gynecology**  **n = 39 (85%)** | **Department**  **Disease specialist n = 7 (15%)** |
| --- | --- | --- |
| Mean age (years) | 38.4 | 40.2 |
| Sex  Male  Female | 8 (20)  31 (80) | 1 (14)  6 (86) |
| Specialism  Gynecologist-perinatologist  Gynecologist-reproductive medicine  Resident gynecology  Midwife  Fertility doctor  Gastroenterologist  Resident gastroenterology  Rheumatologist  Rheumatology consultant | 9 (23)  3 (8)  17 (44)  8 (20)  2 (5)  0 (-)  0 (-)  0 (-)  0 (-) | 0 (-)  0 (-)  0 (-)  0 (-)  0 (-)  2 (29)  1 (14)  1 (14)  3 (43) |
| Registered as specialist  < 1 year  1 – 5 years  > 5 years  Not applicable | 1 (3)  2 (5)  12 (31)  24 (61) | 0 (-)  1 (33)  2 (29)  4 (57) |

* due to missing answers (3.4%) the numbers do not always count up to 100%

**Additional file 7.** Baseline characteristics healthcare professionals.
